# Molecular characterization of foot-and-mouth disease viruses collected from Northern and Central Ethiopia during the 2018 outbreak

**DOI:** 10.14202/vetworld.2020.542-548

**Published:** 2020-03-24

**Authors:** Yeneneh Tesfaye, Fazlurrahman Khan, Esayas Gelaye

**Affiliations:** 1 Department of Biotechnology, School of Engineering and Technology, Sharda University, Greater Noida, Uttar Pradesh, India; 2 Department of Research and Development, National Veterinary Institute, P.O. Box: 19, Bishoftu, Ethiopia; 3 Institute of Food Science, Pukyong National University, Busan 48513, South Korea

**Keywords:** Ethiopia, foot-and-mouth disease virus serotypes, phylogenetic analysis, reverse transcription-polymerase chain reaction

## Abstract

**Background and Aim:**

Foot-and-mouth disease (FMD) is endemic in several developing countries and affects poor farmers through loss of production, death of diseased animals, and loss of animal byproducts. Forty-three samples were collected from 12 sites of five geographical located areas from suspected FMD virus (FMDV)-infected cattle during 2018. This study aimed to isolate and characterize the FMDVs using reverse transcription-polymerase chain reaction (RT-PCR) and gene sequencing.

**Materials and Methods:**

Forty-three FMDV-suspected clinical samples cultured on BHK-21 cell were examined, followed by virus serotype identification using RT-PCR and gene sequencing.

**Results:**

Twenty-nine (67.44%) samples were cultured on BHK-21 cell, of which 14 (32.56%) were not isolated; the 43 samples were analyzed using FMDV screening primers and serotype-specific primers. The contribution of the disease-causing serotype was serotype O of 8 (18.60%) samples, serotype A of 20 (46.51%) samples, and mixed infection (O and A) of 1 (2.33%) sample. Serotypes O and A were further characterized by phylogenetic analysis, which grouped them under East Africa 3 and Africa topotypes of genotype IV, respectively. Interestingly, serotype A was isolated for the 1^st^ time from Keyet sub-woreda and Mulo woreda of Ethiopia, and mixed serotypes (O and A) were identified from the purchased animal.

**Conclusion:**

Molecular test result, sequencing, and phylogenetic tree reconstruction analysis revealed that the 2018 FMD outbreak in Ethiopia was caused by FMDV serotypes O and A. FMDV serotype A was the predominant strain circulating in most study areas of the country. Infections in one sample with mixed serotypes of O and A were also reported. The authors recommend a vaccine matching study of those field isolated viruses with the vaccine strain.

## Introduction

Foot-and-mouth disease (FMD) is a highly contagious viral disease transmitted from animals with open fork leg and is one of the greatest causes of economic and cattle losses [1]. Reports have indicated that there are seven serotypes of FMD viruses (FMDVs) O, A, C, South African Territories [SAT] 1, SAT 2, SAT 3, and Asia 1, which are known to cause diseases [2,3]. The FMDV is the etiological agent of FMD and belongs to the genus *Aphthovirus* and family *Picornaviridae* [4,5]. Based on the sequence analysis of the capsid protein, the serotype is further assigned a topotype, which demonstrates the geographic, antigenic, and genetic relationship among the serotypes [6]. FMD is endemic in Ethiopia, a member of the World Organization for Animal Health (OIE), and the evidence of five of the seven FMDV serotypes have been reported in the country by several research publications [7-9].

Recently, FMD has become the leading cause of blocking the trade of live animals and animal products with Middle Eastern and African countries [1]. Cattle FMDs are among the most critical causes of delaying the growth of the animal industry by reducing animal output and hindering the trade of animal and animal products [7]. Extensive livestock farming, as well as repeated contact at collection points, has become risks factors for FMD in Ethiopia [10,11]. Moreover, the FMD problem in East Africa is intensified by the existence of multiple serotypes [12,13] and the country’s vast wildlife, as well as by the lack of implementation of regulations for controlling the movement of both wildlife and domestic animals within countries and across international borders [8,9,11].

The aim of this study was to isolate and identify different serotypes of the FMDV in cattle from different areas of Ethiopia using a molecular approach. The present study may help the expansion of safe and updated vaccine based on the genetic and antigenic analyses of new viruses isolated from Ethiopia.

## Materials and Methods

### Ethical approval

Ethical approval and consent for this study were obtained from the Ethics and Review Committee of the Addis Ababa University College of Veterinary Medicine and Agriculture Minutes of Animal Research (Reference VM/ERC/01/06/10/2018). In addition, the permission was obtained from animal owners for collecting samples from their cattle and for performing research on the samples.

### Study area and study period

**Figure-1:**
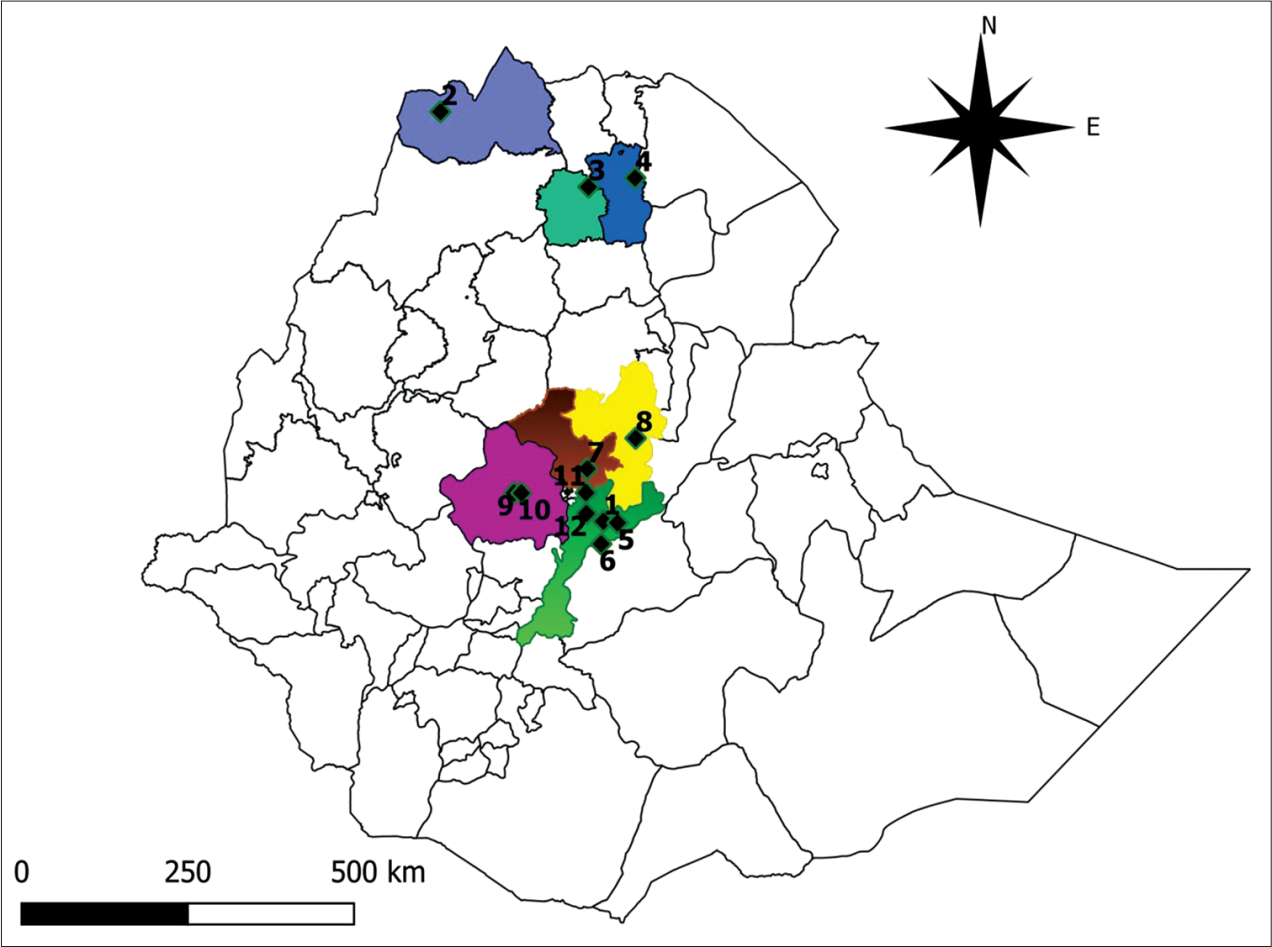
Map of Ethiopia display the location of sample collection sites. Where, 1: Serdo kebele, 2: Asgede-Tsimbila woreda, 3: Kilte-Awulaelo woreda, 4: Hintalo-Wajirat woreda, 5: Denkaka kebele, 6: Jilo-janjo kebele, 7: Jida woreda (Sirte town), 8: Keyet sub-woreda, 9: Tiro-brodorba kebele, 10: Mulo-kersa kebele, 11: Sendafa (kebele 01 and 02), and 12: Debre-zeit kebele 01. [The map was drawn with the help of Quantum GIS 3.10.0 with GRASS 7.8.0 software].

This study was conducted in 12 areas of Ethiopia, where cases of FMD occurred from August to December 2018. The locations of all sampling sites are presented in Figure-1.

### Study design, sample collection, and disease investigation

This study had a cross-sectional design and employed purposive sampling techniques in collaboration with the animal owners and the health service staff at a veterinary clinic [14]. The animals were first calm by restraining techniques for examination and confirmation of clinical symptoms/signs, unbroken and/or ruptured vesicles, erosion, soreness (on the tongue, dental pad, and gum), salivation, and lameness. In total, 43 samples from tongue epithelia, gum tissue, foot tissue, and oral swabs [5] were collected based on the previously mentioned design strategy. The samples were immediately transported through unbroken bottles with cold chain to the Research and Development Department of the National Veterinary Institute (Bishoftu, Ethiopia) for the analysis [15]. The collected samples were also submitted to the World Reference Laboratory for FMD (WRLFMD) in Pirbright (United Kingdom) for sequencing and antigenic variation analysis of the isolates for further comparison with the vaccine strain.

### Virus isolation

The samples were homogenized using sterile mortar and pestle followed by preparing 10% suspension by adding a sterile hanks’ base media. The suspension was clarified through centrifugation at 1500 g for 15 min at 4°C, and the supernatant was collected in universal bottles [11,15]. The confluent monolayer BHK-21 cell was infected with a 0.5 ml tissue suspension that was spread over a cell sheet in a 25 cm^2^ tissue culture flask by tilting for 30 min for better adsorption; thereafter, 10 ml of Hanks’ media was added and the flasks were incubated at 37°C and 5% CO_2_. The cells were examined twice daily under an inverted microscope (TELAVAL3, Germany) until a characteristic cytopathic effect (CPE) was witnessed. The infectious fluid was harvested for 48-72 h post-infection for further molecular analysis [15,16].

Viral RNA was extracted from the original samples and CPE-positive cell culture suspension using the Qiagen RNeasy^®^ Mini Kit (Germany, catalog No. 74106) while following manufacturers’ instructions. Reverse transcription was performed using the Qiagen QuantiNova^™^ Reverse Transcription Kit (Germany, catalog No. 205411) as per instructions. A universal set of primers targeting the FMDV 5′ untranslated region (UTR) (Eurofins Genomics, Austria), FMDV7-For: 5′-GCCTGGTCTTTCCAGGTCT-3′, and FMDV7-Rev: 5′-CCAGTCCCCTTCTCAGATC-3′ was used to screen the samples [17]. Complementary DNA (cDNA) synthesis was conducted in a 20 µl reaction mixture containing 2 µl genomic DNA (gDNA) removal mix, 7 µl template RNA, 1 µl internal control RNA, and 5 µl RNase free water, and the reaction was run at 45°C for 2 min to eliminate gDNA. Thereafter, 5 µl of the cDNA master mix containing 1 µl reverse transcriptase enzyme and 4 µl reverse transcription mix was added for one reaction at 25°C for 3 min (annealing step), 45°C for 20 min (reverse transcription), 85°C for 5 min (inactivation of reaction), and leave at 4°C for infinitive period until it took out from the machine. The cDNA was synthesized in the above running reaction followed by running the polymerase chain reaction (PCR) for FMDV screening. The following are the thermal cycling profiles used for amplification of the 5′ UTR: Initial denaturation at 95°C for 5 min, 35 cycles with denaturing at 95°C for 1 min, annealing at 54°C for 1 min and extension at 72°C for 1 min, and a final extension at 72°C for 5 min. The RT-PCR products (estimated band size of 328 bp) were analyzed through 1.5% agarose gel electrophoresis stained with GelRed (Biotium) for 1 h at 100 V.

The FMDV genome-positive samples were analyzed through a second PCR run for serotyping the O and A virus. Specific primers set FMDVO-For: 5′-CTGCCACCGTCGAGAACTAC-3′ and FMDVO-Rev: 5′-CAGGCGCCACTATCTTCTGT-3′ were used to amplify the 1D gene of serotype O; whereas, FMDVA-For: 5′-TACCAAATTACACACGGGAA-3′ and FMDVA-Rev: 5′-GACATGTCCTCCTGCATCTG-3′ were used amplify the 1C gene of serotype A. The RT-PCR was conducted following a previously described protocol [8,18] with some modification. The thermal cycling profiles used for amplification of the VP1/1D and VP3/1C encoding regions of the serotype O and A viruses were initial denaturation at 95°C for 5 min, 35 cycles with denaturing at 95°C for 1 min, annealing at 58°C for 1 min and extension at 72°C for 1 min 30 sec, and a final extension at 72°C for 7 min except the annealing temperature of 55°C for 1 min for serotype A. The analysis of PCR products with estimated band sizes of 600 bp and 866 bp for serotypes O and A, respectively, was performed through on 1.5% agarose gel electrophoresis stained with GelRed (Biotium) for 1 h at 100 V.

### DNA sequencing

Two independent amplicons, each spanning the entire VP1 region, were generated using the primer sets O-1C244F/EUR-2B52R and O-1C272F/EUR-2B52R for serotype O, and A-1C562F/EUR-2B52R and A-1C612F/EUR-2B52R for serotype A as described in the literature [18]. Thereafter, these were subjected to Sanger sequencing using an ABI 3730 DNA Analyzer (Applied Biosystems) at the Pirbright Institute, United Kingdom [18].

### Phylogenetic analysis

FMDV VP1 gene sequences data were retrieved from the GenBank BLAST database for performing comparative multiple sequence analysis. Using BioEdit, the sequences of the current isolates and reference homologous gene sequences were aligned through multiple sequence alignment with the ClustalW. A phylogenetic tree was constructed using the nucleotide sequences of the present and reference isolates. The neighbor-joining method with the maximum composite likelihood nucleotide substitution model and the pairwise deletion was performed using MEGA version 7.0 [19].

## Results

**Figure-2:**
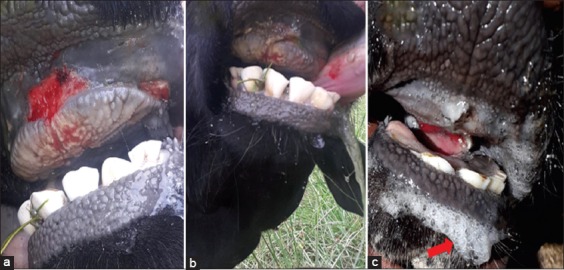
The clinical sign of foot-and-mouth disease. The representative picture was taken during sample collection. (a) Ulceration of gum; (b) Erosion of the tongue; (c) Profuse frothy mouth discharge as indicated in red arrow.

Among the 12 areas investigated for the diseases, animal owners in all except the Bishoftu area had not vaccinated their animals against FMDV. According to oral reports of both Keyet sub-woreda and Jida woreda farmers, newly purchased animals were introduced from local markets before the onset of the FMD outbreak in the above two areas. FMD demonstrated different clinical symptoms in the infected cloven hoof animals, with diverse in harshness between mild and severe. The symptoms of severe form of FMD observed in infected animals included elevated body temperature, vesicular abrasion, and oral soreness. Further, vesicular lesions were witnessed in most of the samples obtained from epithelial tissues (foot, gum, and tongue) of the infected cattle (Figure-2).

**Figure-3:**
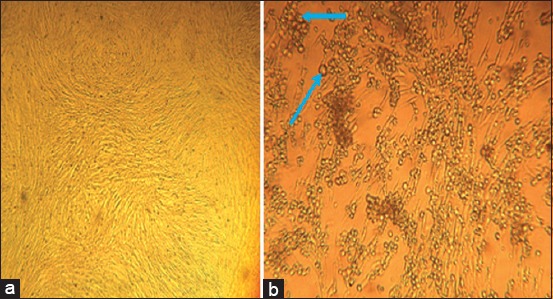
Characteristics of BHK-21 cell culture with and without foot-and-mouth disease virus (FMDV) infection. The representative picture was taken during FMDV isolation. (a) Uninfected (healthy) cell; (b) FMDV infected cell were rounding and cell detachment as indicated in blue arrow.

There are different types of cells used to propagate and adapt FMDV. In this study, we used the BHK-21 cell line to successfully isolate FMDV from the most collected samples. When the virus multiplies in such a cell, it demonstrates its pathogenic effects such as detachment from the culture vessels, aggregate formation, and cell lysis, which release the virus in the culture suspension. Of the total 43 clinical samples, 29 (67.4%) exhibited the CPE specific for FMDV, whereas the remaining 14 (32.56%) samples did not (Figure-3).

**Figure-4:**
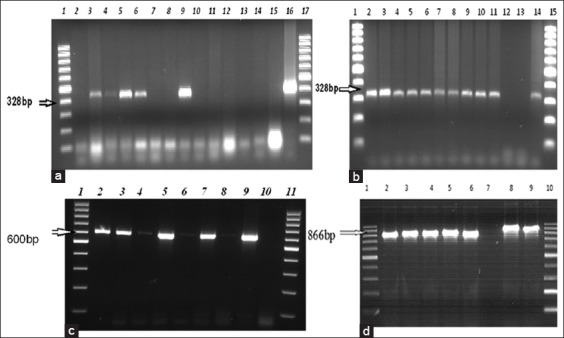
RT-polymerase chain reaction (PCR) amplification for the detection of foot-and-mouth disease virus (FMDV) both from the original and isolated samples. Representative gel electrophoresis picture was taken during FMDV genome screening and typing PCR product of FMDV serotypes O and A. (a) Original samples screening for FMDV shows Lane 1 and 17 of 100 bp DNA ladder, Lane 2, 7, 8, and 10-14 were FMDV negative samples during screen test, Lane 3-6 and 9 were FMDV positive, Lane 15 was negative control, and Lane 16 was positive control during screening (328 bp); (b) isolated samples screening for FMDV shows Lane 1 and 15 of 100 bp DNA ladder, Lane 12 FMDV negative samples during screen test, Lane 13 was negative control, Lane 2-11 were FMDV positive during screening (328 bp); (c) Lane 1 and 11 of 100 bp DNA ladder, Lane 9 was positive control, Lane 10 was negative control, Lane 2-8 were serotype “O” PCR product (600 bp); (d) Lane 1 and 10 were 100 bp DNA ladder, Lane 9 was positive control for serotype A, Lane 7 was negative control for serotype “A,” Lane 2-6 and 8 were serotype “A” PCR product (866 bp).

RNA was extracted from 29 tissue culture fluids tested positive for FMDV in a BHK-21 cell line culture and the original samples, except 14 samples that were not adapted in the cell and lack of FMDV genome. Using FMDV-specific primers, 29 (67.4%) samples were found to be positive for FMDV by RT-PCR (Figure-4). The serotype that causes the disease was serotype O in 8 (18.60%) samples, serotype A in 20 (46.51%) samples, and mixed infection of serotypes O and A in 1 (2.33%) sample; however, the virus genome not detected in 14 (32.56%) of the original samples. Based on the district, all samples originating from five areas Keyet (9/9), Jida (5/5), Asgede-Tsimbla (2/2), Kilte-Awulalo (4/4), and Hintalo-Wajirat (2/2) were found to be 100% positive for FMDV through RT-PCR result; however, samples from Tiro-brodorba (2/3) were 66.67% positive, and those from Sendafa (3/6) and Mulo-Kersa (2/4) were 50% positive. On the contrary, samples from the remaining four sites such as Serdo kebele, Denkaka kebele, Jilo-janjo kebele and Debre-zeit kebele 01 did not indicate the FMDV genome.

**Figure-5:**
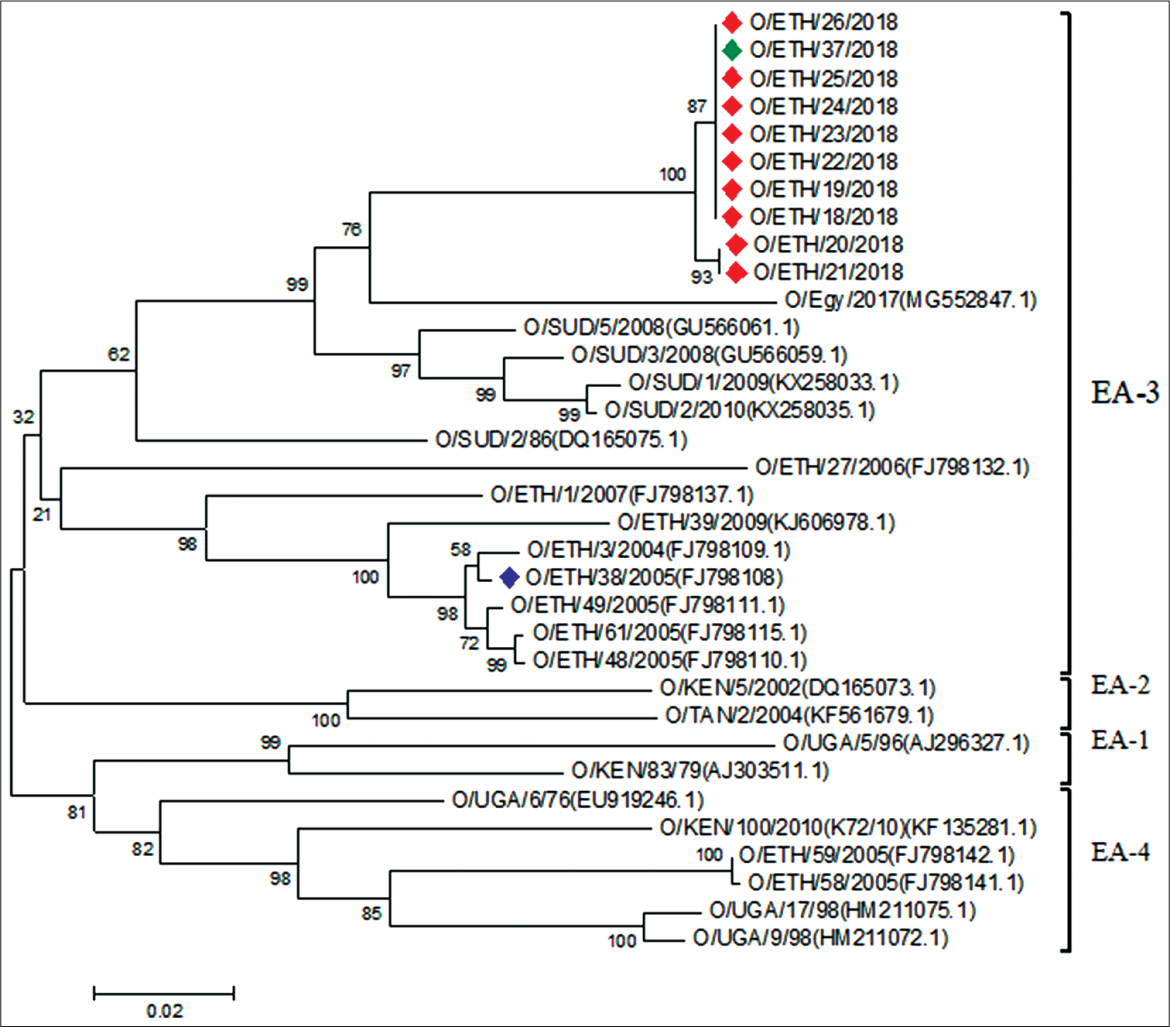
Phylogenetic analysis for the serotype O of foot-and-mouth disease virus. The green diamond label (ETH/37/2018) in the phylogenetic tree was mixed serotypes (O and A) isolated from the purchased calf.

**Figure-6:**
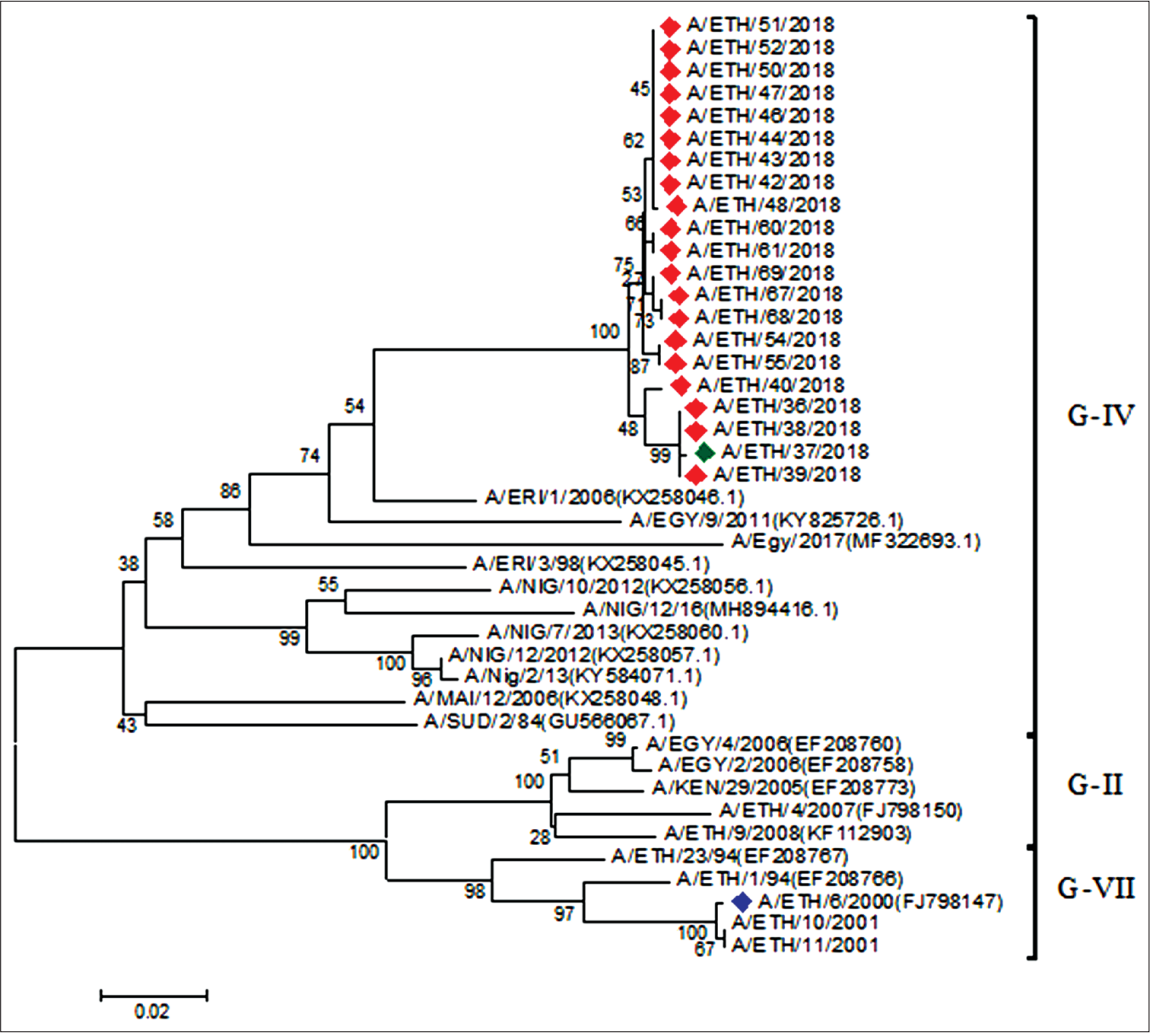
Phylogenetic analysis for the serotype A of foot-and-mouth disease virus. The green diamond label (ETH/37/2018) in the phylogenetic tree was mixed serotypes (O and A) isolated from the purchased calf.

A total of 20 serotype A and 8 serotype O FMDV of the Ethiopian isolates and 1 mixed serotype (O and A) were obtained from the VP1 sequenced encoding gene (Figures-5 and 6). The serotypes O and A from different areas of the country were labeled with a red diamond, while the vaccine strain was labeled with dark blue in the phylogenetic tree followed by a comparison based on the 636 and 633 nucleotide sequences, respectively, of VP1. The VP1 sequences from the three sites were compared to those obtained from the database (Figure-5). The Ethiopian isolates are mostly related to the FMDV from Sudan [O/SUD/5/2008 (GU566061) with 92.02% nucleotide (nt) identity, and O/SUD/2/2010 (KX258035) with 91.08% nt identity and O/SUD/1/2009 (KX258033) with 90.77% nt identity], as well as Egypt [O/Egy/2016 (MF552847) with 90.19% nt identity], which are all classified under the East Africa three (EA-3) topotype.

The VP1 sequence analysis of serotype A isolated from the five sites was compared to those obtained from the GenBank database (Figure-6). The analysis revealed that the Ethiopian isolates are mostly related to the FMDV from Eritrea [A/ERI/1/2006 (KX258046) with 93.10-93.42% nt identity] and Egypt [A/Egypt/9/2011(KY825726) with 89.18-89.66% nt identity], which all classified under the Africa topotype. However, all the fields’ isolate clusters into the same taxa group as indicated in Figure-6 except the vaccine strain A/ETH/6/2000 (FJ798147) labeled in dark blue.

## Discussion

The symptoms observed during sample collection and indicted in the results are in close agreement with the report of Grubman and Baxt [5] and Kandeil *et al*. [20]. Of the 43 samples, 29 were proliferated using BHK-21 cell lines [21]. In the present study, the FMDV-specific CPE was observed, as reported previously by Longjam *et al*. [16], who reported intercellular link, clumping, enlargement, and rounding of the cells. BHK-21 cells can be used as a diagnostic method for viral isolation from oral epithelium [22]; however, pH levels and climatic changes result in decreasing the infectivity of the isolated virus [23]. Our failure to isolate all samples on BHK-21 cell lines may have been because viral isolation depends on live virus, whereas RT-PCR depends on the presence of antigens, either dead or alive virus.

In the present study, mixed serotypes were reported in purchased calf for the 1^st^ time in one sample, which is in conformance with a previous study that found mixed infection by serotype A and Asia-1 in Balochistan, Pakistan [24]. Similarly, a study conducted in Egypt found co-infection of serotypes A/SAT2 and O/SAT2 [25]. Some of the cattle in this study were purchased from a local market Hamuse-gebeya near Sheno town where different animals contact each other. The purchased animals originated from different areas and were brought by a merchant to his home Addis Ababa, where the samples were collected after 6 days when the animal shows the clinical symptom of the disease. This is in alignment with the finding of the previous reports that the spread of FMDV from the carrier and infected animals increases with animal movement [12,13]. Negussie *et al*. [11] stated that the contact between animals at marketing place is a contributed factor for FMD. Based on the serotype basis, FMDV type A was most prevalent in this study followed by type O. In contrast to a previous study, most of the previous outbreaks in Ethiopia were caused by serotype O [7,9] followed by serotype A [8,11]. Serotype A isolates from the five outbreak areas fall under genotype IV. For the 1^st^ time in the above areas, the FMDV was grouped under the Africa topotype and genotype IV, in alignment with previous Sulayeman *et al*. [14] report from the Arsi, Guna, and Kolfe areas falls into genotype IV and Africa topotype [6]. In samples from the three outbreak areas, serotype O falls into the East Africa topotype 3 [6,8,25] and is the predominant lineage of the virus in this country [8].

## Conclusion

The present study confirms that the serotypes O and A were predominant spread of FMDV in Ethiopian during August-December 2018. The conventional RT-PCR for capsid protein amplification followed by nucleotide sequence analysis was confirmed that the serotype A was dominant over serotype O. The 1^st^ time mixed infection having serotypes O and A was also detected in young calf which was purchased from the market in the nation. The isolated virus now could be used for the expansion of effective vaccine production at NVI, Ethiopia, after conducting amino acid analysis in critical sites of the capsid and vaccine matching study against the currently used vaccine.

## Authors’ Contributions

YT, EG, and FK designed the experiment. YT collected samples and performed the experiment. All authors were involved in the writing, analysis of the data, and reviewed the manuscript, and they approved the final manuscript.
